# Tranilast administration reduces fibrosis and improves fatigue resistance in muscles of *mdx* dystrophic mice

**DOI:** 10.1186/1755-1536-7-1

**Published:** 2014-01-30

**Authors:** Kristy Swiderski, Michelle Todorov, Stefan M Gehrig, Timur Naim, Annabel Chee, David I Stapleton, René Koopman, Gordon S Lynch

**Affiliations:** 1 Basic and Clinical Myology Laboratory, Department of Physiology, The University of Melbourne, Melbourne, Victoria 3010, Australia

## Abstract

**Background:**

Duchenne muscular dystrophy (DMD) is a severe and progressive muscle-wasting disorder caused by mutations in the dystrophin gene that result in the absence of the membrane-stabilising protein dystrophin. Dystrophic muscle fibres are susceptible to injury and degeneration, and impaired muscle regeneration is associated with fibrotic deposition that limits the efficacy of potential pharmacological, cell- and gene-based therapies. Novel treatments that can prevent or attenuate fibrosis have important clinical merit for DMD and related neuromuscular diseases. We investigated the therapeutic potential for tranilast, an orally bioavailable anti-allergic agent, to prevent fibrosis in skeletal muscles of *mdx* dystrophic mice.

**Results:**

Three-week-old C57Bl/10 and *mdx* mice received tranilast (~300 mg/kg) in their food for 9 weeks, after which fibrosis was assessed through histological analyses, and functional properties of tibialis anterior muscles were assessed *in situ* and diaphragm muscle strips *in vitro*. Tranilast administration did not significantly alter the mass of any muscles in control or *mdx* mice, but it decreased fibrosis in the severely affected diaphragm muscle by 31% compared with untreated *mdx* mice (*P* < 0.05). A similar trend of decreased fibrosis was observed in the tibialis anterior muscles of *mdx* mice (*P* = 0.10). These reductions in fibrotic deposition were not associated with improvements in maximum force-producing capacity, but we did observe small but significant improvements in the resistance to fatigue in both the diaphragm and TA muscles of *mdx* mice treated with tranilast.

**Conclusion:**

Together these findings demonstrate that administration of potent antifibrotic compounds such as tranilast could help preserve skeletal muscle structure, which could ultimately increase the efficacy of pharmacological, cell and gene replacement/correction therapies for muscular dystrophy and related disorders.

## Background

Duchenne muscular dystrophy (DMD) is a severe, X-linked genetic muscle-wasting disorder characterised by progressive muscle weakness that culminates in respiratory failure and premature death. The disease affects approximately 1:3,500 live male births worldwide, and affected boys are usually wheelchair bound by their early teens and experience a severely reduced quality of life. DMD is caused by mutations in the dystrophin (*dmd*) gene resulting in very low levels or a complete absence of the dystrophin protein, a key structural element of muscle fibres that renders them highly susceptible to damage. As a consequence, dystrophic muscles are characterised by inflammation and ongoing cycles of degeneration and regeneration. This environment limits muscle regenerative capacity and there is concomitant replacement of formerly functional muscle fibres with adipose and fibrotic material (reviewed in [1]).

The *mdx* mouse is the most commonly used animal model of DMD. It arose from a natural mutation in a colony of C57BL/10 mice in which a premature stop codon was introduced into the *dmd* gene, resulting in a complete loss of the dystrophin protein [2,3]. While this model is a good genocopy of the human disease, the pathology of the *mdx* mouse does not mimic that observed in DMD. *Mdx* mice exhibit a mild pathology with a slightly reduced lifespan (18–24 months) and functional decline of hindlimb muscles becoming evident at approximately 18 months of age [4], which is most likely a result of modifier genes in the C57BL/10 strain that can alter the effect of dystrophin loss on skeletal muscle pathology. Unlike the hindlimb muscles, the diaphragm of *mdx* mice undergoes progressive deterioration of muscle structure and function and is therefore a preferred muscle when examining the efficacy of potential therapeutic agents of clinical relevance [5-7].

As DMD is caused by mutations in a single gene, one of the most promising therapies is through gene replacement. However, while gene replacement or correction studies are likely to provide an eventual cure for DMD, several barriers need to be overcome including the presence of fibrosis within dystrophic skeletal muscles (reviewed in [8]). Fibrosis not only creates a physical barrier, but also replaces the muscle fibres that can be targeted, limiting the efficacy of cell- and gene-based therapies. Attenuating fibrotic infiltration may be needed to optimise gene, cell and pharmacological therapies.

Various agents with antifibrotic properties have been trialled to reduce fibrosis deposition in skeletal muscle. Suramin, a TGF-β inhibitor, and interleukin-15 (IL-15) have been shown to reduce muscle fibrosis but can have side effects when administered systemically [9-11]. Another compound with antifibrotic properties is tranilast, an orally bioavailable antiallergic agent that has been approved for use in the human population in Japan and South Korea since 1982 for the treatment of bronchial asthma, atopic dermatitis and allergic rhinitis [12-17]. Since that time, the effectiveness of tranilast as a therapeutic agent for a range of fibrotic disorders and its mechanism of action have been studied extensively both *in vitro* and *in vivo* (reviewed in [18]). In 1992, Suzawa and colleagues [19] demonstrated that tranilast suppressed release of profibrotic cytokines from monocyte-macrophages *in vitro*[19], highlighting tranilast’s antifibrotic properties. Tranilast has subsequently been demonstrated to reduce tuberointerstitial and heart fibrosis in diabetic rat models and to block TGF-β-induced fibrosis *in vitro* and *in vivo*[20-24]. In addition, tranilast administration was found to be efficacious in reducing muscle fibrosis in the Bio14.6 hamster model of limb-girdle muscular dystrophy and reducing serum creatine kinase levels in *mdx* dystrophic mice, effects they suggest may be a result of tranilast-mediated inhibition of the Ca^2+^-permeable growth factor-regulated channel (TRPV2 or GRC) [18,25]. In this study, we report that short-term administration of tranilast in *mdx* mice decreases fibrosis in skeletal muscle and improves the resistance to muscle fatigue. Together these findings demonstrate that tranilast has therapeutic potential to combat fibrosis in muscle diseases such as DMD.

## Results

### Tranilast does not alter skeletal muscle mass or strength

**Figure 1:**
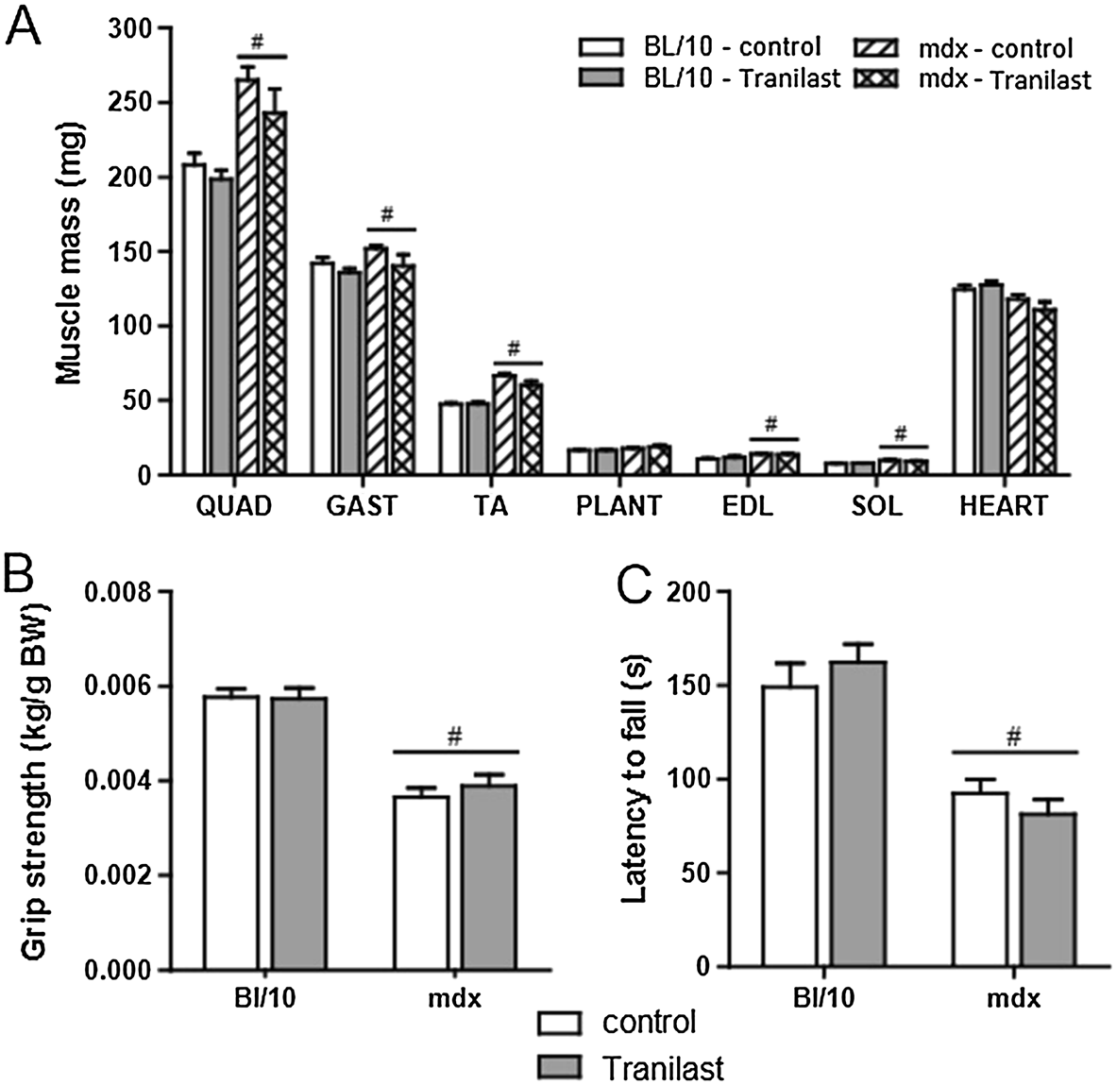
**Tranilast effects on muscle mass and whole body strength of *mdx *mice.** Three-week-old male C57Bl/10 control mice or mdx mice received either vehicle or tranilast treatment for 9 weeks. **(A)** Individual muscle masses at the end of the treatment period. **(B)** Grip strength was measured 3 days prior to endpoint. **(C)** Whole body function and coordination was assessed by rotarod performance 2 days prior to endpoint. QUAD: quadriceps; GAST: gastrocnemius; TA: tibialis anterior; PLANT: plantaris; EDL: extensor digitorum longus; SOL: soleus. ^#^*P* < 0.05 group main effect, mdx vs. control.

At the end of the 9-week treatment period, the tibialis anterior (TA), soleus (SOL), extensor digitorum longus (EDL), plantaris (PLANT), gastrocnemius (GAST), quadriceps (QUAD) and heart muscles from both non-treated and treated *mdx* mice were significantly larger than those from non-treated and treated control mice (Figure 1A). These differences cannot be attributed to differences in food intake as daily intake was not different between strains or treatment groups (data not shown) and averaged 3.3 g/mouse/day. Administration of tranilast did not significantly alter the mass of any of the tested skeletal muscles in control or *mdx* mice (Figure 1A). Consequently, 9-week treatment with tranilast did not affect whole-body strength (Figure 1B) or mobility (Figure 1C), as assessed by grip strength and rotarod performance.

### Fibrotic deposition is decreased in muscles of tranilast-treated dystrophic mice

**Figure 2:**
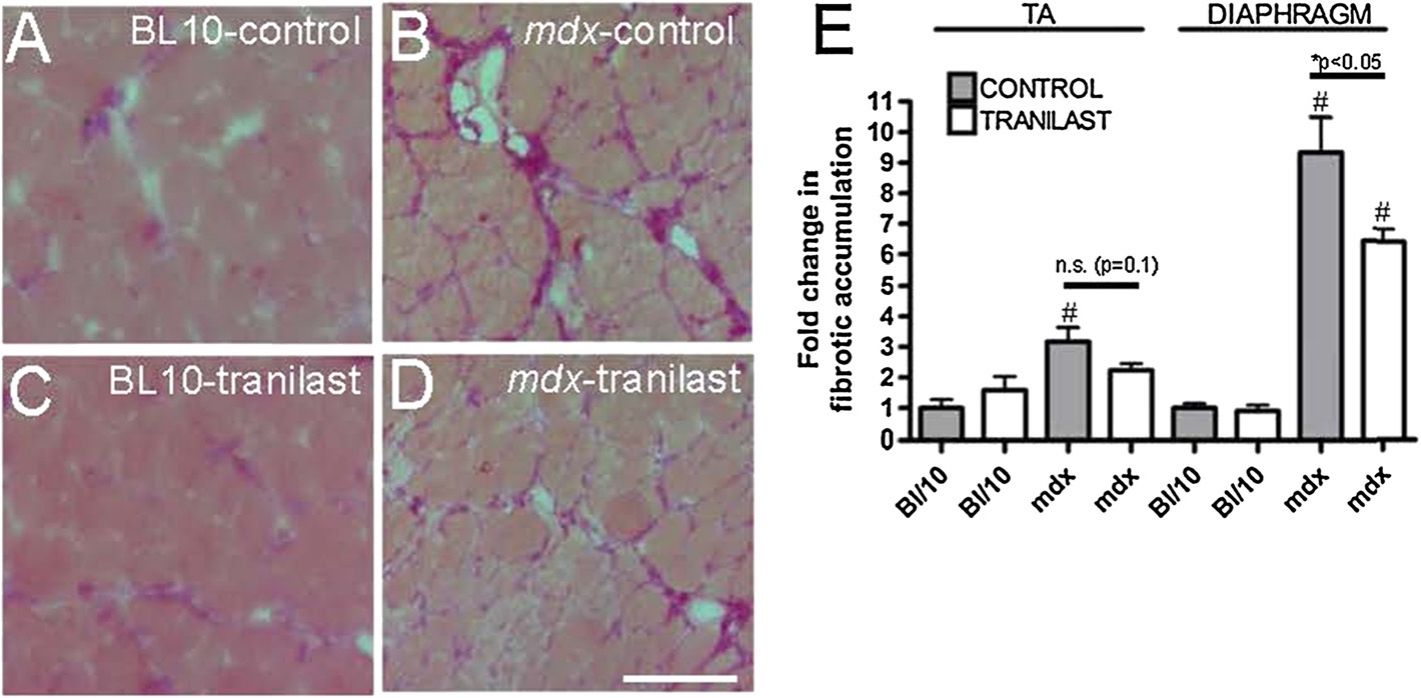
**Tranilast effects on fibrosis in the diaphragm and TA of *mdx *mice.** At endpoint, the diaphragm muscle of vehicle-treated C57BL/10 **(A)**, vehicle-treated mdx **(B)**, tranilast-treated C57BL/10 **(C)** or tranilast-treated mdx **(D)** mice was excised and collagen infiltration visualised in cross-sections after Van Gieson’s staining. **(E)** Fibrotic accumulation in Van Gieson’s stained sections from diaphragm and tibialis anterior muscles of untreated and treated mice. ^#^P < 0.05 group main effect, mdx vs. control. *P < 0.05 mdx treated vs. mdx control; *n.s*, not significant.

The TA and diaphragm muscles of *mdx* mice contained three- and nine-fold more fibrosis, respectively, compared with control mice (Figure 2). Tranilast administration to young *mdx* mice for 9 weeks resulted in a significant three-fold (31%) decrease in fibrosis in the diaphragm compared with untreated *mdx* mice (Figure 2A-C, *P* < 0.05). A similar trend (30% decrease, *P* = 0.10) was observed in the TA muscles of *mdx* mice. The level of fibrosis in the TA muscles and diaphragm of control animals was naturally very low and unchanged with tranilast administration (Figure 2C). Analysis of gene expression revealed that Collagen 1a1, 3a1, 5a1 and TGFβ genes were all more highly expressed in the diaphragm of *mdx* compared to C57BL/10 mice, but tranilast treatment had no effect on the expression levels of these genes (Table 1). Tranilast treatment resulted in a change in fibre type distribution in the TA muscles of *mdx* mice with an increased proportion of type IIa fibres with a concomitant decrease in type IIb/x fibres compared with muscles from untreated *mdx* mice (Table 2). No significant differences were observed between tranilast-treated and control *mdx* mice in fibre cross-sectional area or oxidative enzyme capacity in either the TA or diaphragm muscles (Table 2).

**Table 1:** Tranilast administration does not cause a long-term change in fibrotic gene expression in the diaphragm

	**CON + VEH**	**CON + TRA**	***mdx-*** **VEH**	***mdx-TRA***
**Col1a1**	1.00±0.09	0.83±0.13	3.42±0.42*	3.37±0.33*
**Col2a1**	1.00±0.28	1.23±0.47	0.79±0.20	0.92±0.34
**Col3a1**	1.00±0.10	0.89±0.13	3.72±0.26*	3.27±0.34*
**Col4a1**	1.00±0.06	0.87±0.11	1.31±0.14	1.35±0.13
**Col5a1**	1.00±0.09	0.91±0.08	3.46±0.24*	3.40±0.36*
**COl6a1**	1.00±0.06	0.87±0.11	1.31±0.14	1.35±0.13
**Fn1**	1.00±0.14	1.34±0.07	1.00±0.14	1.01±0.18
**TGFβ1**	1.00±0.11	0.86±0.07	1.48±0.04*	1.45±0.12*

At the conclusion of the treatment period RNA was extracted and real-time PCR analysis was used to examine the expression level of genes known to be associated with fibrosis. *P < 0.05 *mdx* vs. control.

**Table 2:** **Tranilast administration causes a shift in fibre type proportions in the TA muscles of *mdx *** **mice**

	**CON + VEH**	**CON + TRA**	***mdx*** **+ VEH**	***mdx*** **+ TRA**
**Tibialis anterior**				
**Fibre CSA (μm**^**2**^**)**	1,811.4 ± 54.9	1,859.7 ± 74.3	1,890.2 ± 54.0	1,932.2 ± 113.6
**SDH intensity (o.d.)**	1,645.4 ± 57.4	1,622.3 ± 36.6	1,497.2 ± 74.1^†^	1,464.7 ± 48.9^†^
**% Type IIa fibres**	11.0 ± 3.1	9.1 ± 1.6	4.9 ± 0.6	10.8 ± 0.5*
**Diaphragm**				
**Fibre CSA (μm**^**2**^**)**	1,010.6 ± 69.3	949.8 ± 69.8	764.9 ± 39.9^†^	874.6 ± 65.3^†^
**SDH intensity (o.d.)**	2,004.0 ± 63.9	1,725.6 ± 212.3	1,897.3 ± 46.2^†^	1,793.1 ± 124.1^†^
**% Type IIa fibres**	63.8 ± 1.6	67.8 ± 3.9	71.5 ± 2.6^†^	78.6 ± 3.3^†^

At the conclusion of the treatment period the fibre cross-sectional area (CSA), fibre type and oxidative enzyme activity were determined by histological evaluations of cross-sections stained with H&E and reacted for succinate dehydrogenase (SDH) activity. ^†^*P* < 0.05, group main effect; *P < 0.05, tranilast-treated vs. vehicle-treated *mdx* mice.

### Tranilast administration improves resistance to muscle fatigue in dystrophic mice

**Figure 3:**
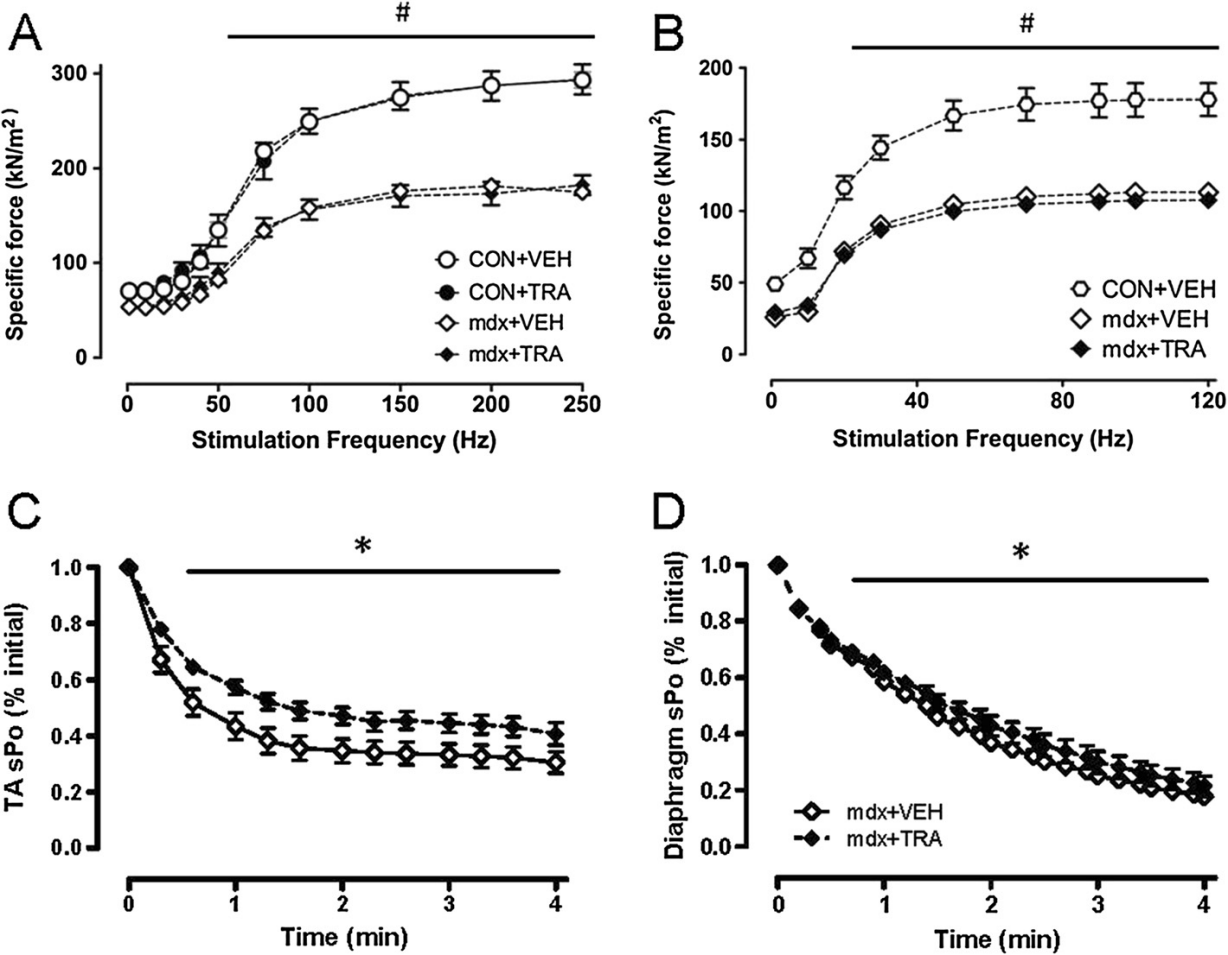
**Tranilast effects on fatigue of diaphragm muscle strips and tibialis anterior muscles of *mdx *mice.** Analysis of the frequency-force relationship showed significant reductions in specific force in the *mdx* TA and diaphragm compared to controls, which was not altered with administration of tranilast **(A/B)**. At the conclusion of treatment, resistance to fatigue was determined from intermittent stimulation of **(C)** diaphragm muscle strips evaluated *in vitro* or **(D)** TA muscles evaluated *in situ*, from vehicle or tranilast-treated *mdx* mice. Values are expressed as relative specific force (force per cross-sectional area) during the 4-min stimulation protocol. ^#^*P* < 0.05 group main effect, *mdx* vs. control. *P < 0.05 treatment main effect.

Dystrophic *mdx* mice exhibited a ~40% reduction in diaphragm- and TA-specific force compared with control (Figure 3A/B). Nine-week treatment with tranilast did not improve whole-body strength or mobility and did not improve maximum force-producing capacity in the TA or diaphragm muscles of control or *mdx* mice (i.e., for TA muscle: C57BL/10 control 1,825 ± 35 mN vs. C57Bl/10 tranilast 1,743 ± 55 mN; *mdx* control 1,456 ± 88 mN vs. *mdx* tranilast 1,177 ± 139 mN). However, force production during a 4-min fatiguing stimulation protocol was improved in both the diaphragm and TA muscles of tranilast-treated *mdx* mice (Figure 3B, C).

### Tranilast impairs glucose tolerance in control and dystrophic mice

**Figure 4:**
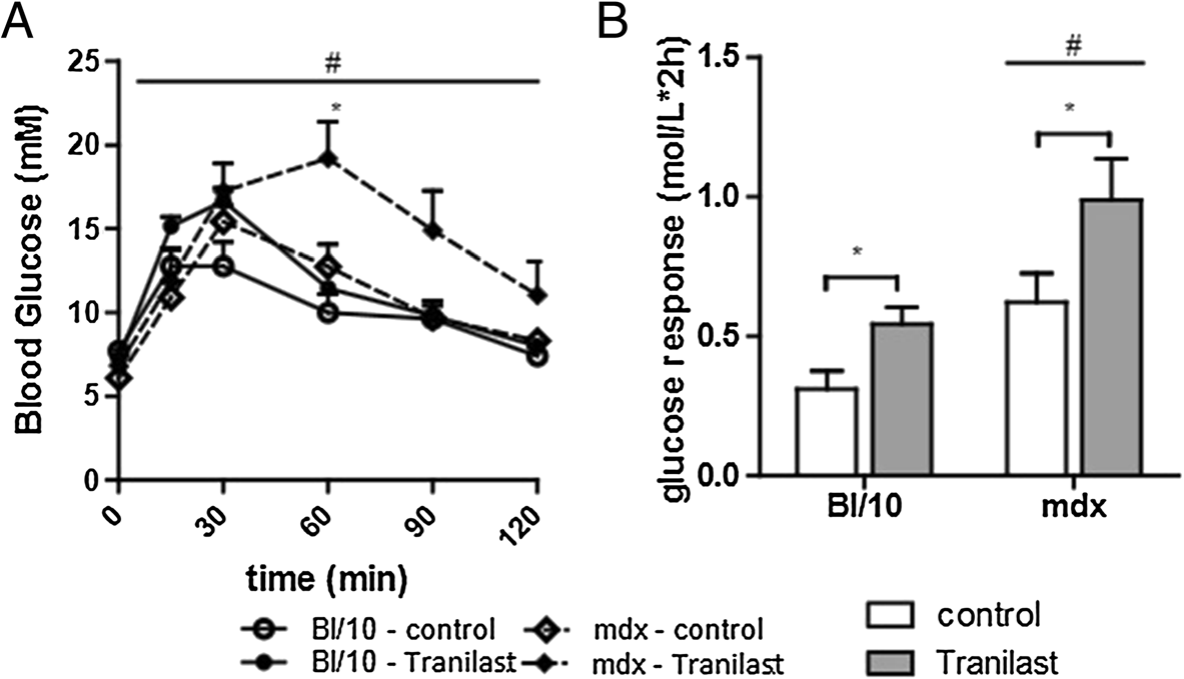
**Tranilast treatment effects on glucose tolerance in *mdx *mice.** Glucose tolerance tests were performed on vehicle and tranilast-treated control and *mdx* mice during the final week of treatment. Changes in blood glucose over time during the glucose tolerance test **(A)** and calculated glucose response **(B**; area under the curve). ^#^*P* < 0.05 group main effect, *mdx* vs. control. *P < 0.05 treatment main effect.

To check whether tranilast administration altered glucose handling in control and dystrophic mice we also performed a glucose tolerance test. Dystrophic *mdx* mice exhibited impaired glucose tolerance as evidenced by a 100% higher glucose response following a single intraperitoneal injection of glucose (1 g/kg) (expressed as area under the curve, Figure 4, *p* < 0.05). While basal blood glucose levels were not affected by tranilast administration, ~20% increased peak blood glucose levels were observed in treated control and *mdx* mice compared with untreated mice during the GTT (Figure 4). Furthermore, the blood glucose response was ~70% higher in tranilast-treated control and *mdx* mice compared with untreated mice (Figure 4).

## Discussion

The identification of pharmacological agents that can prevent, reduce and/or resolve fibrotic deposition has great potential for enhancing therapies for DMD and other muscle-wasting disorders. While gene and cell therapies will eventually provide the cure for the single-gene muscle-wasting disorders, the efficacy of these approaches is likely to be hampered by the presence of significant fibrosis within affected skeletal muscles. Here we have demonstrated that one agent, tranilast, successfully reduces fibrotic deposition in skeletal muscles of *mdx* dystrophic mice.

Tranilast has been administered to sarcoglycan-deficient Bio14.6 hamsters, a rodent model of limb-girdle muscular dystrophy (LGMD). Treatment of 30-day-old hamsters for 120 days significantly decreased fibrosis in skeletal muscle and reduced serum creatine kinase levels and the number of centrally nucleated muscle fibres, indicating reduced muscle fibre breakdown and regeneration [25]. That study also observed a reduction in serum creatine kinase levels after a 30-day treatment in 30-day-old *mdx* mice [25]. We have subsequently demonstrated that oral administration of tranilast (~300 mg/kg) to young mice for 9 weeks significantly reduced fibrotic accumulation by ~30% in the diaphragm muscles of *mdx* mice (Figure 2A, B, C). We observed a similar trend towards a decrease in fibrosis accumulation in the TA muscles of treated *mdx* mice but this was not statistically significant (*P* = 0.10). This is most likely due to the low levels of fibrosis in the TA muscles compared with those in the diaphragm of *mdx* mice. The observed decrease in the diaphragm, which is the most severely affected of the muscles in the *mdx* mouse, indicates that tranilast was able to reduce fibrotic accumulation. We did not observe a change in the maximum force production of the TA muscle or diaphragm muscle strips, which is not surprising since a 30% reduction in fibrosis in these muscles of *mdx* mice would only increase the available functional tissue in the TA and diaphragm by 1.5% and 5%, respectively. We did not observe an improvement in whole-body strength or mobility of the treated mice or in maximum force of diaphragm muscle strips. Other compounds with specific muscle effects and antifibrotic properties such as IL-15 and sildenafil have been shown to improve the muscle force-producing capacity [26,27]. However, we did observe an improvement in the fatigability of both the diaphragm and TA muscles in treated *mdx* mice, indicating a potential improvement in muscle function. This improved fatigue resistance was not due to any changes in the muscle oxidative capacity, as there were no differences in muscle fibre oxidative capacity (SDH) between control and tranilast-treated mice (Table 1).

Although tranilast administration successfully decreased fibrotic tissue infiltration in dystrophic skeletal muscle, one concern was that it also resulted in impaired glucose tolerance in both dystrophic and control mice. Tranilast has been shown to inhibit insulin secretion in rats [28], so long-term treatment may not be possible using this specific compound. However, the effective human dose of tranilast for fibrotic pathology has been shown to be 5 mg/kg, and subsequent safety information regarding the use of tranilast in humans at this dose has not indicated toxicity issues associated with effects on insulin secretion [18]. It remains to be determined whether this dose, which is significantly lower than the dose used in the present study, would be sufficient to alter fibrosis in human skeletal muscle. Therefore it is possible that newer generation drugs may be required for therapeutic application. To this end, more targeted drugs based on the structure of tranilast are being developed that can attenuate interstitial fibrosis in the hearts of diabetic rats without causing hyperglycaemia [29].

## Conclusion

Interventions to minimise fibrosis are important not just for skeletal muscle diseases but also to enhance functional recovery after serious muscle injuries. Although tranilast decreased fibrosis in dystrophic skeletal muscles, functional benefits were limited to modest improvements in fatigue resistance with impaired glucose tolerance also being a limiting factor. These issues need to be overcome in order to improve the therapeutic relevance and efficacy. Together these findings demonstrate that administration of potent antifibrotic compounds such as tranilast and newer drugs could help preserve skeletal muscle structure to ultimately increase the efficacy of pharmacological, cell and gene replacement/correction therapies for muscular dystrophy and related disorders.

## Methods

### Animals

Three-week-old male C57BL/10 (*n* = 32) and C57BL/10ScSn *mdx* (*mdx*) (*n* = 32) mice were obtained from the Animal Resources Centre (ARC), WA, Australia. All experimental protocols were approved by the Animal Ethics Committee of The University of Melbourne and conducted in accordance with the Australian code of practice for the care and use of animals for scientific purposes as stipulated by the National Health and Medical Research Council (Australia).

Mice were allocated into one of four groups: (1) control group treated with vehicle (CON + VEH, *n* = 16); (2) control group treated with tranilast (CON + TRA, *n* = 16); (3) *mdx* group treated with vehicle (*mdx* + VEH, *n* = 16); (4) *mdx* group treated with tranilast (*mdx* + TRA, *n* = 16).

### Tranilast administration

Three-week-old control (C57BL/10) and *mdx* mice received tranilast (kindly provided by Dr. Spencer Williams, Department of Chemistry, The University of Melbourne) for a period of 9 weeks. Mice received standard laboratory chow (Speciality Feeds, Glen Forrest, WA, Australia) with or without the addition of 2.7 g tranilast/kg. Food was made available *ad libitum* based on the assumption that mice would consume approximately 4 g of feed per day resulting in a dose of 400 mg/kg/day of tranilast. This dose has been shown previously to reduce collagen infiltration in heart [20] and kidney [30] of diabetic rats. Food intake was monitored throughout the treatment period [20] and as mice consumed an average 3.3 g/day, daily tranilast intake averaged 293 mg/day.

### Measurement of whole body strength

Whole body strength, whole body mobility and coordination were assessed in control C57BL/10, treated C57BL/10, control *mdx* and treated *mdx* mice at 2 (mobility) or 3 (body strength) days prior to endpoint by means of a grip strength meter (Columbus Instruments, Columbus, OH) and rotarod performance test (Rotamex-5, Columbus Instruments) as described previously [31].

### Glucose tolerance test

Glucose tolerance tests were performed on control C57BL/10, treated C57BL/10, control *mdx* and treated *mdx* mice 5 days prior to endpoint. Following an overnight fast, a basal blood sample was taken from the tail vein (23 G needle) and analysed for glucose concentration using a glucometer. Mice then received an intraperitoneal injection of glucose solution (1 g/kg body mass). At 15, 30, 60, 90 and 120 min after the injection of the glucose solution, a blood sample was collected from the tail vein (23 G needle) and analysed for glucose concentration.

### Assessment of contractile properties of skeletal muscle and tissue collection

At the conclusion of the treatment period, mice were anesthetised with sodium pentobarbitone (Nembutal; 60 mg/kg; Sigma-Aldrich) *via i.p*. injection. The methods for assessment of the contractile properties of the mouse tibialis anterior (TA) muscle *in situ* have been described in detail previously [32]. At the conclusion of the contractile measurements *in situ*, the TA muscle was carefully excised, blotted on filter paper and weighed on an analytical balance, followed by freezing in thawing isopentane for later histological examination. Soleus (SOL), extensor digitorum longus (EDL), plantaris (PLAN), gastrocnemius (GAST) and quadriceps (QUAD) muscles were excised, blotted on filter paper, trimmed of their tendons and adhering tissue, weighed and frozen in liquid nitrogen.

The entire diaphragm and rib cage were then surgically excised and costal diaphragm muscle strips composed of longitudinally arranged full-length muscle fibres were isolated and prepared for functional assessment *in vitro*, as described in detail elsewhere [32]. Upon completion of the functional analyses, the diaphragm muscle strip was trimmed of tendon and any adhering non-muscle tissue, blotted once on filter paper and weighed on an analytical balance. The muscle strips were then frozen in thawing isopentane for later histological examination. Mice were killed as a consequence of diaphragm and heart excision while deeply anesthetised.

### Skeletal muscle histology and fibrosis

Serial sections (5 μm) were cut transversely through the diaphragm and the TA muscle using a refrigerated (-20°C) cryostat (CTI Cryostat; IEC, Needham Heights, MA). Sections were stained with haematoxylin and eosin (H&E) to reveal the general muscle architecture. Muscle fibre cross-sectional area, oxidative enzyme capacity and fibre type were determined as described previously [33]. Briefly, TA and diaphragm sections were reacted histochemically for succinate dehydrogenase (SDH) activity and immunoreacted with antibodies to laminin (Sigma-Aldrich) and myosin IIa, N2.261 (The University of Iowa, Department of Biology, Iowa City, IA; USA) in order to determine the oxidative capacity, CSA of individual myofibers and proportion of type IIA fibres respectively. Muscle collagen content was assessed from Van Gieson-stained cross-sections [32]. Digital images of stained sections were obtained using an upright microscope with a camera (Axio Imager D1, Carl Zeiss, Wrek, Göttingen, Germany), controlled by AxioVision AC software (AxioVision AC Rel. 4.8, Carl Zeiss Imaging Solutions, Wrek, Göttingen, Germany). Images were quantified using AxioVision 4.8 software.

### Analysis of gene expression

At the conclusion of the treatment period, diaphragm muscles were excised and total RNA was extracted using a commercially available kit, according to the manufacturer’s instructions (PureLink RNA Mini Kit, Invitrogen). The RNA concentration was determined by a spectrophotometer at 260 nm and subsequently transcribed into cDNA using the Superscript VILO cDNA synthesis kit (Invitrogen). Real-time RT-PCR was performed as described previously [34] using the following forward and reverse primer sequences: Col1a1, 5′-CACCCTCAAGAGCCTGAGTC-3′and 5′-GTTCGGGCTGATGTACCAGT-3′; Col2a1, 5′-GCCAAGACCTGAAACTCTGC-3′ and 5′-GCCATAGCTGAAGTGGAAGC-3′; Col3a1, 5′-ACCAAAAGGTGATGCTGGAC-3′ and 5′-GACCTCGTGCTCCAGTTAGC-3′; Col4a1, 5′-AAAGGGAGAAAGAGGCTTGC-3′ and 5′-CCTTTGTACCGTTGCATCCT-3′; Col5a1, 5′-GGTCCCTGACACACCTCAGT-3′ and 5′-TGCTCCTCAGGAACCTCTGT-3′; Col6a1, 5′-CCCCATTGGACCTAAAGGAT-3′ and 5′-TCTCCCACTTCACCCTCATC-3′; Fn1, 5′-ACCACCCAGAACTACGATGC-3′ and 5′-GGAACGTGTCGTTCACATTG-3′; TGFβ1, 5′- TGAGTGGCTGTCTTTTGACG-3′ and 5′- TCTCTGTGGAGCTGAAGCAA-3′. Gene expression was quantified using a cycle threshold (C_T_) method, whereby a ΔC_T_ was calculated by subtracting the 18S C_T_ from the gene C_T_. The relative gene expression was then calculated using the expression 2^-ΔCT^.

### Statistical analyses

Data were analysed with the GraphPad Prism software. Statistical significance was determined using a two-way analysis of variance (ANOVA; for comparison of treatment and group), with significance set at *P* < 0.05. A Tukey post hoc multiple comparison test was used where appropriate to determine significance between groups. For fatigue data comparing multiple time points, a two-way repeated measures ANOVA was used. Values are presented as mean ± SEM.

## Abbreviations

CSA: Cross-sectional area; DMD: Duchenne muscular dystrophy; EDL: Extensor digitorum longus; GAST: Gastrocnemius; LGMD: Limb girdle muscular dystrophy; mdx: Muscular dystrophy X-linked; PLAN: Plantaris; QUAD: Quadriceps; SOL: Soleus; TA: Tibialis anterior; TGFβ: Transforming growth factor-β; TRA: Tranilast; VEH: Vehicle.

## Competing interests

None of the authors have any competing interests.

## Authors’ contribution

KS, SMG, DIS, RK and GSL conceived and designed the experiments. MT, KS, AC and TN performed the experiments. KS, MT, SMG, DIS, RK and GSL have analysed and interpreted the data. KS, MT, DIS, RK and GSL have drafted and revised the manuscript. All authors have given final approval of the latest version of the manuscript.
